# Stewardship Intervention to Optimize Central Venous Catheter Utilization in Critically Ill Children

**DOI:** 10.1097/pq9.0000000000000389

**Published:** 2021-02-12

**Authors:** Jennifer A. Blumenthal, Jennifer A. Ormsby, Dimple Mirchandani, Chonel A. Petti, Jane Carpenter, Maggie Geller, Stephanie N. Harding, Mary O’Brien, Thomas J. Sandora, Monica E. Kleinman, Gregory P. Priebe, Nilesh M. Mehta

**Affiliations:** From the * Department of Anesthesiology, Critical Care and Pain Medicine, Division of Critical Care Medicine, Boston Children’s Hospital, Boston, Mass.; † Department of Pediatrics, Division of Infectious Diseases, Boston Children’s Hospital, Boston, Mass.; ‡ Harvard Medical School, Boston, Mass.; § Infection Prevention and Control, Boston Children’s Hospital, Boston, Mass.

## Abstract

We aimed to describe utilization and indication(s) for long-term central venous catheters (CVCs) in a pediatric intensive care unit (PICU) and identify potential strategies to decrease CVC utilization.

**Methods:**

We conducted a single-center prospective quality improvement initiative at a 30-bed PICU in a large, freestanding, academic children’s hospital. We created an electronic report to identify patients with an indwelling CVC for 7 days and older (defined as long term). We discussed the ongoing need for each long-term CVC with PICU clinicians at weekly interdisciplinary structured “CVC stewardship rounds.” We then made recommendations around expedited removal of CVCs. We conducted multiple Plan-Do-Study-Act cycles to categorize CVC indications, identify modifiable factors, and educate PICU clinicians. We hypothesized that CVC stewardship rounds would decrease long-term CVC utilization in our PICU.

**Results:**

From October 2016 to September 2017, 607 long-term CVCs were eligible for the stewardship intervention. Compared to the preintervention period, we recorded a significant increase in peripherally inserted central catheters and a decrease in nontunneled CVCs (*P* < 0.001). Most patients had single- or double-lumen CVCs in both the preintervention and intervention periods (86% and 91%, respectively). The utilization of overall long-term CVC devices, and those with modifiable indications, decreased during the intervention period.

**Conclusions:**

A single-center QI intervention focused on PICU CVC stewardship was associated with a decrease in CVC utilization.

## INTRODUCTION

Central line-associated bloodstream infection (CLABSI) is one of the leading healthcare-associated infections in the intensive care unit (ICU). It is associated with increased morbidity, length of stay, and hospital costs.^1,2^ The incidence of CLABSIs and their impact on patients remain significant despite advances in infection prevention strategies.^3,4^ Bundled insertion and maintenance plans improve CLABSI rates.^5,6^ The Centers for Disease Control and Prevention (CDC) guidelines for best practices provide recommendations for insertion site disinfection, dressing types and frequency, tubing change, and antimicrobial-coated catheters.^7^ Also, the guidelines review educational modeling, which serves as the basis of CLABSI prevention.

Pediatric-specific guidelines for central venous catheter (CVC) insertion and maintenance are based on scarce scientific literature than adult guidelines. Pediatric ICU clinicians have developed consensus-based bundles to lower infection rates in their population and meet national patient safety goals set by the Joint Commission.^8^ Interventions aimed at assessing the ongoing need for CVCs in pediatric intensive care unit (PICU) patients, specifically long-term CVCs, have not been adequately described in the literature. Long-term CVCs (*>*7 d dwell time) are at a significantly higher risk for infectious and noninfectious morbidities than short-term CVCs.^5,7,9,10^ Additionally, a CLABSI incidence of 26.2% for idle CVCs (not medically indicated) has been described in the transition from the adult ICU to the inpatient wards.^11^ Quality improvement (QI) efforts aimed at reduction of CVC days by eliminating idle CVCs and minimizing modifiable indications are desirable.^4,7^ The discussion of CVC need, with an effort to encourage thoughtful use and timely removal, are critical elements of “CVC stewardship.”

In our PICU in 2015, CVC utilization rates were higher than the 90th percentile compared with those in other centers participating in the National Healthcare Safety Network (NHSN).^3^ Despite multipronged approaches targeted at improving compliance for insertion and maintenance bundles, our center’s mean for CLABSI rates remained unchanged around 1.96 CLABSI/1,000 CVC days. A different approach, targeted at optimizing CVC indications and decreasing CVC indwelling time, seemed necessary. We aimed to establish a novel CVC stewardship intervention to accomplish 2 aims: (1) to understand current CVC utilization and identify potentially modifiable practices, and (2) to decrease unnecessary/modifiable CVC use and reduce total CVC days. We hypothesized that there would be a decrease in CVC utilization (CVC days/patient days) after implementing the stewardship intervention.

## METHODS

The project was a single-center QI initiative. We de-identified patient-specific data and described summary/group results as trends. According to institutional policies, this project was exempt from IRB review.

Boston Children’s Hospital is a 404-bed pediatric academic institution that is Magnet designated. The 30-bed pediatric Medical–Surgical ICU (the “PICU”) has over 2,000 admissions annually. Major diagnostic categories include neurology/neurosurgery, oncology, bone marrow transplant, and complex medical and surgical diagnoses. The PICU QI committee consists of a QI director, consultant, and champions representing physicians, nursing, respiratory therapists, pharmacists, trainees, and administrative staff. Based on the prevailing rates of CLABSI in the PICU, the QI committee determined the need for an innovative approach to reduce the CLABSI rate. The multidisciplinary committee identified this intervention as a priority effort and included it in the PICU quality management plan. A subset of the PICU QI Committee conducts weekly QI walk rounds to engage with frontline providers using a structured process. The “CVC stewardship rounds” described in this article are now part of the weekly QI walk rounds.

**Fig. 1.**
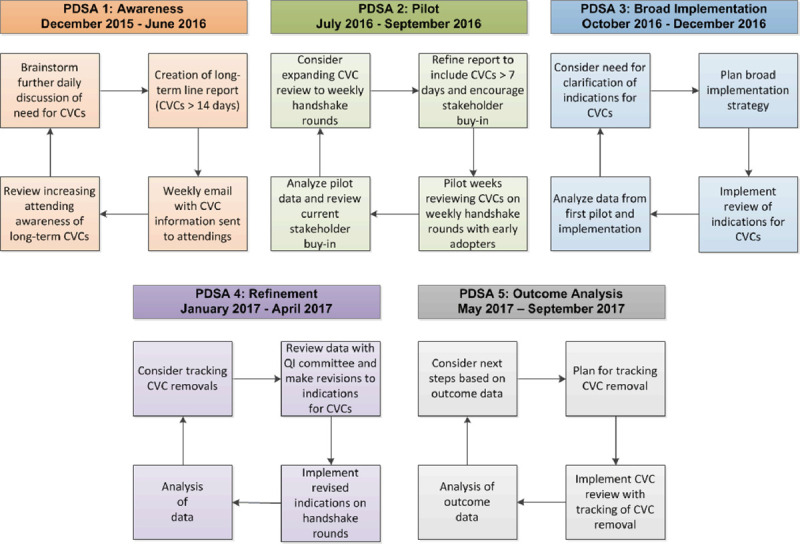
PDSA cycles overview for the stewardship project.

We employed the Model of Improvement Plan-Do-Study-Act (PDSA) cycle to test small changes in short cycles.^12^ Figure 1 outlines our 5 PDSA cycles (called “cycles” for brevity).

The main focus of the project included:

1. six months of increasing awareness for providers using data to demonstrate the current practice and discuss modifiable practice;
2. just-in-time data to guide discussion and decisions around ongoing CVC use;
3. one-on-one discussions with providers, including attending physicians, to understand barriers to best practice in individual cases; and
4. modifying subsequent cycles based on the information and evaluating each cycle’s cumulative impact on CVC practice.

*Cycle 1*: Our first PDSA cycle aimed to raise awareness and educate providers on the prevalence and risks of long-term CVC use in our PICU. With informatics support, we created an automated report that listed all patients with long-term CVCs (14 d or longer)^13^ and the number of days the CVCs had been in place. We sent this report to attending physicians at the start of their week of service to increase awareness of long-term CVCs. To maximize the detection of eligible CVCs, we subsequently amended the definition to include all CVCs that had been in place for 7 days or longer. With the PICU and QI committees’ leadership support, we started our CVC stewardship rounds with a small group of attending providers.

*Cycle 2*: The second PDSA cycle involved discussing each long-term CVC (7 d or longer) with the patient’s attending provider on a subset of teams and collecting reasons for ongoing CVC use. Options for CVC indications included high patient acuity as defined by the provider, multiple sedative infusions (such as analgesia/sedative medications), need for parenteral nutrition, difficulty obtaining peripheral venous access, or other as described by the clinician. We categorized the indications for CVC usage as modifiable or nonmodifiable a priori. A modifiable reason included any case where an alternative to CVC was deemed feasible by the stewardship group. We also documented if the CVC was no longer needed, and the treating team intended to remove the line.

*Cycle 3*: As part of this PDSA cycle, we implemented the “CVC stewardship” rounding with all providers in October 2016. During weekly QI rounds, the QI team discussed each long-term CVC with the patient’s PICU attending (or designee if the attending was unavailable). The attending would select the most appropriate indication(s) for the CVC in that patient. We documented when a CVC was deemed unnecessary by the attending and followed up to provide a reminder in 72 hours if necessary.

*Cycle 4*: Our fourth PDSA cycle involved further refinement of our indications into more specific categories and labeling the indications as either potentially modifiable or nonmodifiable on the day of discussion. We challenged the notion of definite or nonmodifiable indications for CVC during this cycle and used discussions to educate and support providers for alternatives to CVC when deemed available. Nonmodifiable indications included: “acute” patient, multiple continuous infusions for analgesia/sedative medications, parenteral nutrition (PN), long-term chemotherapy, dialysis/pheresis, and others. In these situations, removing the CVC would result in the patient’s inability to receive essential care. Patients with a potentially modifiable indication included those in whom CVC removal was planned, the need for antibiotic administration, the patient awaiting a planned procedure, and provider preference. The stakeholders’ consensus was that these patients could receive essential care with a peripheral intravenous (IV) catheter. We deemed difficulty obtaining peripheral venous access a nonmodifiable indication only after exploring the help from the institutional IV placement team. If the CVC indication was potentially modifiable, the QI team discussed possible options that would enable the provider to remove the CVC.

*Cycle 5*: During the fifth PDSA cycle, we added a list of active medications for each patient to provide a memory tool for improving the completeness of the indications in our documentation. We shared this list with the care team during the weekly CVC stewardship rounds. We also added a report indicating the total number of times per 24-hour period that each CVC was accessed. We included this report to improve general awareness both of lines that were being infrequently accessed (and therefore potentially could be removed) and frequently accessed lines to assure each time was necessary (given the risk of introduction of bacteria associated with each access) and to evaluate potential medication changes from IV to enteral.

We enhanced buy-in from providers by ensuring the participation of unit leadership in CVC stewardship rounds. We provided succinct and efficient information and adapted rapidly to requests or suggestions for team members’ workflow improvement. We added a report of the number of CVC days and the number of CVC entries per day for each long-term CVC in the fifth PDSA cycle based on feedback. The weekly CVC stewardship rounds lasted between 25 and 30 minutes by the end of cycle five.

**Fig. 2.**
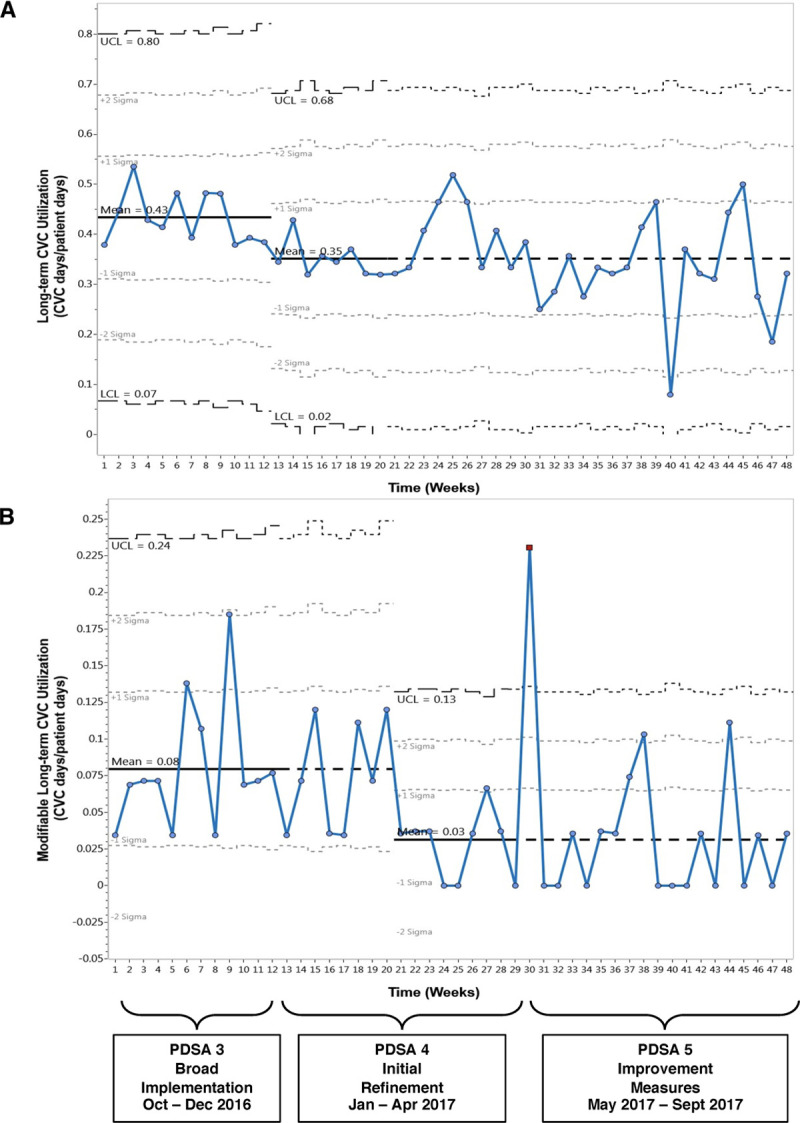
Process control charts showing trends in CVC utilization in the PICU.

**Fig. 3.**
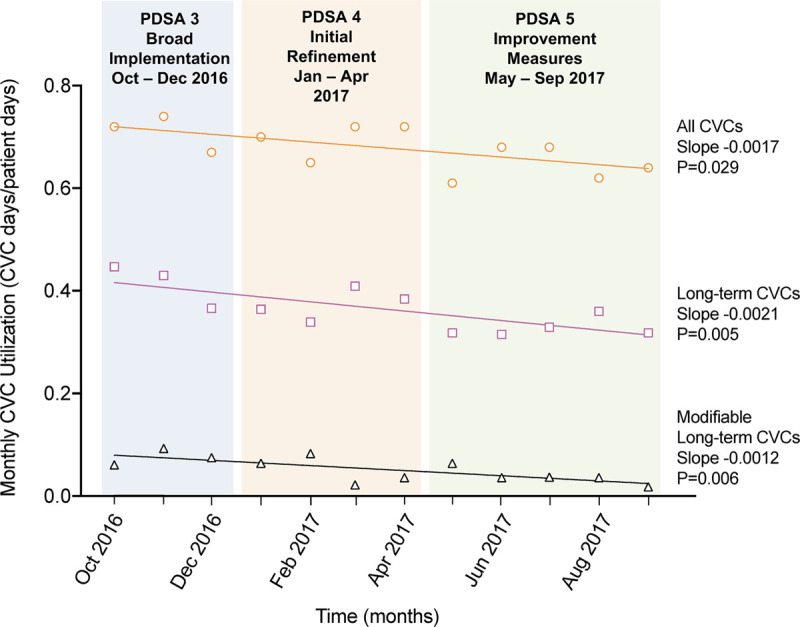
Temporal trends of the utilization of CVCs in the PICU. A, all CVCs. B, Long-term CVCs evaluated during the stewardship intervention. C, Long-term CVCs with a modifiable indication.

We analyzed the data using Microsoft Excel, SQC Pack QI software (version number 7.0.18187.5; Dayton, Ohio), and GraphPad Prism (version number 8.2.1; San Diego, Calif.). The primary outcome measures included utilization rates of long-term CVCs (defined as the number of CVCs with 7 days or longer dwell time divided by the number of patient days) and modifiable CVCs during the intervention period. We also assessed weekly trends in the utilization rates for all CVCs during the intervention period compared to the preperiod. We employed the CDC/NHSN definition of CVC utilization. The numerator of each point in Figure 2 is the number of patients with one or more long-term CVCs (or modifiable CVCs) on the day of rounds, and the denominator is the number of patients in the unit on that day. In Figure 3, these are grouped monthly for the long-term and modifiable CVCs.

In contrast, the “All CVCs” label in Figure 3 refers to the monthly CVC days/patient days calculated by NHSN definitions where every day of the month is included. Modifiable indications for CVC continuation were a priori defined by the QI committee and infection control team. We created and analyzed statistical process control charts for the 2 outcome measures to assess our CVC stewardship intervention’s effects over time.^14^ We examined these data for any evidence of special cause variation, using standard definitions. We used linear regression (GraphPad Prism) to determine whether the slopes of the trendlines of CVC utilization rates were significantly different from zero. Our process measure was the percent of long-term CVCs reviewed per week; this was 100% on each of the 50/52 weeks of conducted CVC stewardship rounds. To compare CVC characteristics, we completed a chart review of all long-term CVCs for the 12 months, October 2015 to September 2016 (the “preperiod”). We used the chi-squared test and Fisher’s exact test to determine the statistical difference between these characteristics for the 2 periods. Patient characteristic data for both the preperiod and the intervention period were collected to evaluate potential confounders or unintended consequences. Patient characteristics included mortality rates, Pediatric Index of Mortality 3 (PIM3) severity scores, and ICU length of stay.

## RESULTS

Table 1 details demographic information from the preperiod and CVC stewardship intervention period. Over the year of CVC stewardship intervention, 607 CVCs met the inclusion criteria, compared to the 556, which would have been eligible for intervention in the year prior (preintervention). Diagnostic categories included 31.4% surgical patients in the preperiod and 22.9% in the intervention period, and 41.3% neurology/neurosurgery patients in the preperiod versus 42.2% in the intervention period. There were no statistically significant differences between the median ages, PIM3 scores, mortality, or diagnostic categories between the 2 groups. Median ICU length of stay was also similar between the 2 groups.

**Table 1.** Demographic and Clinical Characteristics of Patients with One or More CVC for 7 Days or Longer in a Pediatric Medical–Surgical ICU

Characteristic		Pre-CVC Stewardship Evaluation Period October 1, 2015–September 30, 2016	CVC Stewardship Evaluation Period October 1, 2016–September 30, 2017
		No. unique patients qualifying for evaluation (n = 223)	No. unique patients evaluated (n = 192)
Median age in years (range)		3.7 (0–30.1)	2.4 (0–28.3)
Male sex		110 (49.3%)	115 (59.9%)
Average PIM3 generated probability of death (range)		1.3% (0.3–5.5%)	1.5% (0.4–6.3%)
No. CLABSI		11	11
Dx	Surgical	70 (31.4%)	44 (22.9%)
	Medical	18 (8.1%)	20 (10.4%)
	Neuro (surgical/medical)	92 (41.3%)	81 (42.2%)
	Oncology	25 (11.2%)	20 (10.4%)
	HSCT	18 (8.1%)	27 (14.1%)
ICU LOS (d)	Median (range)	21 (0.2–253.7)	17.5 (0.7–205)
Mortality		13 (5.8%)	16 (8.3%)

Dx, diagnostic category; HSCT, hematopoietic stem cell transplant.

Table 2 describes the CVC characteristics for the preintervention period (based on the chart review) and the intervention period. Peripherally inserted CVCs (PICCs) made up the majority of the CVCs in each period, which increased from the preperiod to the intervention period from 54% to 67% (*P* < 0.001). In comparison, temporary nontunneled CVCs decreased from 27% in the preintervention period to 13% in the intervention period (*P* < 0.001). Most patients had single- or double-lumen CVCs in both the pre and intervention periods (86% and 91%, respectively). The indication for insertion, which is selected by the provider at the time of insertion, was similar for “current access unable to support treatment” but increased in the “infusion requirement” category from 27% to 36% (*P* < 0.002). Also, the proportion of CVCs retained for long-term chemotherapy decreased from the preperiod (30%) to the intervention period (20%). As shown in Table 3, 88.5% of the long-term CVC indications were nonmodifiable, whereas 11.5% were potentially modifiable.

**Table 2.** CVC Characteristics in Patients with One or More CVC for 7 Days or Longer

CVC Characteristic		Preperiod (n = 556)	Intervention Period (n = 607)	*P*
CVC types	PICC	298 (54%)	404 (67%)	<0.001
	Tunneled, cuffed device	66 (12%)	103 (17%)	0.014
	Temporary, nontunneled CVC	150 (27%)	76 (13%)	<0.001
	Umbilical catheter	4 (<1%)	1 (<1%)	0.2
	Hemodialysis/pheresis	37 (7%)	23 (4%)	0.027
CVC vein type	Internal jugular/SVC	117 (21%)	80 (13%)	<0.001
	Femoral	61 (11%)	43 (7%)	0.020
	Subclavian	67 (12%)	72 (12%)	0.921
	Peripherally inserted	305 (55%)	411 (68%)	<0.001
	Other (including umbilical and portal)	6 (1%)	1 (<1%)	0.06
No. CVC Lumens	One	73 (13%)	110 (18%)	0.020
	Two	407 (73%)	445 (73%)	0.966
	Three	76 (14%)	52 (9%)	0.005
CVC insertion location	All intensive care units	203 (37%) 167	290 (48%)	<0.001
	MSICU	(30%)	268 (44%)	<0.001
	MICU	11 (2%)	7 (1%)	0.255
	NICU	10 (2%)	7 (1%)	0.340
	CICU	15 (3%)	8 (1%)	0.091
	OR/procedural unit	277 (50%)	190 (31%)	<0.001
	Outside hospital	10 (2%)	71 (12%)	<0.001
	Interventional radiology	58 (10%)	44 (7%)	0.055
	General floors	8 (1%)	12 (2%)	0.481
Indications for CVC insertion*	Current access unable to support treatment, or lack of peripheral veins to support treatment	347 (33%)	363 (33%)	0.9
	Dialysis/pheresis	27 (3%)	32 (3%)	0.61
	Hemodynamic monitoring	49 (5%)	31 (3%)	0.03
	Home parenteral therapy	16 (2%)	34 (3%)	0.02
	Infusion requirement (vasoactive infusion, infusion hyperosmolality, pH < 5 or > 9)	288 (27%)	392 (36%)	<0.002
	Long-term chemotherapy	311 (30%)	216 (20%)	<0.001
	Solid organ transplant	1 (<1%)	4 (<1%)	0.38
	Stem cell transplant	13 (1%)	16 (2%)	0.37

*Denominator for this section is total indications. Many patients have multiple indications for insertion documented, all included.

CICU, cardiac intensive care unit; MICU, medical intensive care unit; MSICU, medical surgical intensive care unit; NICU, neonatal intensive care unit; OR, operating room; SVC, superior vena cava.

**Table 3.** Provider-specified Indications for Ongoing CVC

Nonmodifiable	537 (88.5%)
Acute patient	192 (31.6%)
PN	104 (17.1%)
Sedation	73 (12%)
Multiple continuous infusions	72 (11.8%)
Long-term chemotherapy	45 (7.4%)
Sedation and PN	31 (5.1%)
Dialysis/pheresis	13 (2.1%)
Other	7 (1.1%)
Potentially modifiable	70 (11.5%)
None: planned removal	28 (4.6%)
Difficult access	20 (3.3%)
Antibiotics	10 (1.6%)
Provider preference	6 (1%)
Preprocedure	6 (1%)

Figure 2A and B shows the process control charts for weekly utilization of long-term CVCs and CVCs deemed modifiable starting in PDSA cycle 3 when modifiable CVCs were fully defined. The weekly control charts show that the centerline for long-term CVC utilization shifted downward early in PDSA cycle 4 (from 0.43 to 0.35 CVC days/patient days, a 19% relative decrease). The centerline for modifiable CVCs shifted downward 2 months later, in the middle of PDSA cycle 4 (from 0.08 to 0.03 CVC days/patient days, a > 50% relative decrease). Both of the new centerlines were maintained at their lower levels through the rest of the intervention period. However, the modifiable CVC utilization rate had a single point above the upper control limit early in PDSA cycle 5 that was of a unclear cause. Figure 3 compares the trendlines of monthly CVC utilization for long-term CVCs, long-term CVCs with modifiable indications, and all CVCs in the PICU (the typical NHSN-reportable metric) starting in PDSA cycle 3. All 3 trendlines show statistically significant downward trends in CVC utilization during the intervention period. Notably, in the preperiod from October 2015 to September 2016, the monthly utilization trend had a slope that was not significantly different from zero (slope −0.00045, *P* = 0.59). The median duration of modifiable CVCs was 43 days (interquartile range [IQR] 63) in the first 6 months of the intervention (October 2016 to March 2017) compared to a shorter duration of 32 days (IQR 66) in the second 6 months (April 2017 to September 2017).

## DISCUSSION

The PICU QI team chose to target the meaningful use of CVCs, explicitly aiming to optimize the indications and eliminate avoidable continuation of indwelling CVCs in the PICU, termed “CVC stewardship.” In particular, we targeted the duration of the use of long-term CVC intending to decrease overall long-term CVC days in the PICU. Following a multifaceted QI initiative that included transparency, increased awareness, and direct in-person discussions, we demonstrated a significantly decreasing CVC utilization trend. The project resulted in a culture that promoted proactive discussion related to the need for CVCs, clarifying reasonable indications for continued utilization of CVCs in patients, exploring other options, and considering the replacement of percutaneous CVCs with PICCs or tunneled CVCs.

Continued use of CVCs beyond the absolute minimum necessary duration is a risk factor for CLABSI. However, there are not well-documented efforts to target CVC duration to decrease CLABSI rates in the literature, particularly in pediatrics. In a small study of adult patients in Thailand, the authors reported a statistically significant decrease in the number of CVC days following an intervention that promoted physician documentation of the indication for the ongoing use of CVC.^15^ In another recent study from the United States in an adult long-term acute care facility, weekly multidisciplinary infection prevention team rounds evaluating CVCs communicated recommendations for removal to the primary physician, resulting in nonsignificant improvement in CLABSI rates over the study timeframe of four months, without the ability to sustain the change.^16^ Multiple studies focus on daily reminders of the need for a CVC and use various approaches for delivering reminders, including multidisciplinary teams. All of these studies noted improvement in CVC days and statistically significant decreases in CLABSI rates.^17–19^ To our knowledge, data on such interventions in pediatric institutions are not available.

The number of long-term CVCs in our PICU is high, and the indications for ongoing use are usually related to sedation, nutrition, or patient acuity. Given the overall decrease in the CVCs with potentially modifiable indications, especially in combination with the unchanging total CVCs over time, we believe our effort was successful. Despite our PICU already having high compliance with the documented daily discussion of CVC need, we found that at least 10% of CVCs had potentially modifiable indications. Education around daily discussion of need should continue.

One must interpret the results of our QI initiative in light of some limitations. Our single-center experience may not be generalizable to other units with a different structure, size, or patient population. The indications documented for the CVC at the time of placement were at the provider’s discretion and not always complete. For example, there may have been more than one indication (eg, PN and difficult vascular access), although only one indication may have been documented. Also, the modifiable and nonmodifiable groups were a priori determined by the CVC stewardship team, and improvements in those definitions over subsequent cycles have clarified the terminology. Unlike in-person interviews with providers during the intervention period, we relied on a chart review for the preintervention period, which may have less accurately recorded all the indications for CVC use. The lack of assessment of balancing measures (eg, whether a new CVC was required within 24 h of recommended removal) is another limitation. Last, we were unable to collect the duration of CVCs for the nonmodifiable CVCs given the lack of clarity on the timing of removal in the electronic medical record, so we cannot address the total CVC duration for all central lines, which could help clarify the effect of our interventions.

There were also strengths to our process, including the multiple iterations and the ability to include provider feedback directly over multiple PDSA cycles. Local leadership support and their presence at the front lines of this stewardship intervention were pivotal factors in the success of this QI initiative. The variability among our patient population and provider population, including 3 distinct medical teams, helped ensure a more generalizable group of patients than might be seen in a more focused unit such as a cardiac ICU. The detailed information collected and the prospective nature of CVC indications were also beneficial in truly understanding in real-time what was driving the modifiable indications and how we could enhance education to improve the removal of idle CVCs.

## CONCLUDING SUMMARY

We have demonstrated a positive influence of our QI effort on CVC utilization. Our results emphasize the importance of a multidisciplinary QI effort that includes leveraging electronic health records to promote data-driven awareness, practice modifications, and outcome improvement. The proactive discussions around the actual need for ongoing use of CVCs may help eliminate some of the barriers to the timely removal of CVCs in the PICU population. Ideally, in the future, we would explore the impact of information technology on reducing healthcare-associated infections in busy and complex healthcare systems.

## DISCLOSURE

The authors have no financial interest to declare in relation to the content of this article.

## ACKNOWLEDGMENTS

This quality improvement project was funded, in part, by the Department of Anesthesiology, Critical Care and Pain Medicine’s Division of Critical Care Medicine funds for quality improvement. We acknowledge the ongoing commitment to patient safety and quality improvement of Boston Children’s Hospital and the Medical/Surgical Intensive Care Unit, whose Quality Improvement Committee remains dedicated to improving patient safety.
